# Eye movement patterns correlate with overt emotional behaviours in rapid eye movement sleep

**DOI:** 10.1038/s41598-022-05905-5

**Published:** 2022-02-02

**Authors:** Jean-Baptiste Maranci, Milan Nigam, Luc Masset, Eva-Flore Msika, Marie Charlotte Vionnet, Charlotte Chaumereil, Marie Vidailhet, Smaranda Leu-Semenescu, Isabelle Arnulf

**Affiliations:** 1 Sleep Disorder Unit, Pitie-Salpetriere University Hospital, APHP, Paris, France; 2 Center for Advanced Research in Sleep Medicine, Hôpital du Sacré-Coeur de Montréal, Montréal, Canada; 3 Paris Brain Institute, Paris, France; 4 Sorbonne University, Paris, France

**Keywords:** Emotion, Neurological disorders

## Abstract

Growing evidence suggests that sleep plays a key role in regulating emotions. Rapid eye movements (REMs) in REM sleep could be associated with dreams emotions, but supporting evidence is indirect. To highlight this association, we studied the REM sleep during video-polysomnography of 20 subjects with REM sleep behaviour disorder (RBD), a model of enacted dreams offering direct access to the emotional content of the sleeper (face expression, speeches, behaviour). Video and the electro-oculography recordings were divided into 3 s time intervals and classified as non-behavioural, or behavioural (neutral, positive or negative emotions), and as containing no eye movements (EMs), slow eye movements (SEMs) or REMs (isolated or bursts). Compared to the absence of EMs, neutral behaviours successively increased in the presence of SEMs (odd ratio, OR = 1.4), then isolated REMs (OR = 2.8) and then REM bursts (OR = 4.6). Positive behaviours increased with SEMs (OR = 2.8) but did not increase further with isolated REMs (OR = 2.8) and REM bursts (OR = 3). Negative behaviours were absent with SEMs, increased with isolated REMs (OR = 2.6) and further with REM bursts (OR = 10.1). These results support an association between REMs and SEMs, and dream emotions.

## Introduction

Why do we have rapid eye movements (REMs) during REM sleep? Although this question has intrigued researchers since their discovery in 1953, its answer remains elusive. Interest in this question has been reinforced by the discovery of an increase in REMs during REM sleep in patients suffering from depression^1^. This REMs excess appears to be present even in at-risk subjects who have not yet started depression, which suggests that it may be an endophenotypic aspect of the disease^2^. A better understanding of REMs could thus provide insight into the mechanisms leading to depressive disorder.

The "scanning" hypothesis (the most studied so far) postulates that eye movements during REM sleep follow dream scenery (for a review of evidence for and against this hypothesis, see Arnulf)^3^. It has also been proposed that REMs are associated with the reactivation of emotional memories in dream^4,5^. This hypothesis is consistent with postulated role of dream-associated emotions in emotional regulation^6^. The empirical evidences favouring this hypothesis come from studies using functional MRI, EEG, magnetoelectroencephography (MEG), and intracranial surface EEG, and converge towards showing an increased activation of the amygdala at the time of the REMs^7–12^. As the amygdala is a key structure associated with emotions during wakefulness, its time activation with REMs may support the theory of emotional reactivation during REM sleep^13^. However, inferring emotional content from amygdala activation during REMs alone is problematic, because the amygdala is a complex structure also involved in non-emotional functions^14^. Moreover, REMs are also associated with the simultaneous activation of several other brain areas, including the occipital cortex, motor cortex, as well as language areas, which are not specifically associated with emotional process^15,16^. Plus, dream reports from patients with Urbach-Wiethe disease (a rare genetic disorder associated with bilateral dysfunction of the basolateral amygdala) were unexpectedly emotional, and positive emotions were even more common than in the dream reports of healthy subjects^17^. However, a decrease in the intensity of negative emotions could be observed in patients with Urbach-Wiethe disease and dream reports were shorter and simpler than in healthy subjects, thus not completely excluding the influence of the amygdala on dreaming. One may access to the genuine emotions felt by sleeping subjects when they are reported after awakening and look back if they are exclusively or more frequently associated to REM sleep epochs with (phasic REM sleep) vs. without REMs (tonic REM sleep). This method is inherently problematic however, as dream reports can be biased by an imperfect recall of dreams and the chronological sequences of dream scenery. Thus, reported dreams may not actually correspond to dreams occurring immediately prior to awakening.

One way to circumscribe limitations of dream reports is to use the REM sleep behaviour disorder (RBD) as a model. This disorder is characterized by dream enactment caused by the loss of REM sleep muscle atonia, which results in patients exhibiting purposeful behaviours in concordance with the dream reports obtained upon awakening^18,19^. Patients with RBD smile, laugh, shout, cry, fight, speak with emotional tone and display overt negative and positive facial expressions translating the ongoing emotion^20,21^. As such, RBD constitutes a unique window allowing to observe the dreaming process (at the very moment it occurs) and its neurophysiological markers from an external, objective point of view. Using this model, we aimed at studying whether eye movements (EMs) are temporally associated with overt facial expressions and behaviours which relate the emotions of the sleeper.

## Results

**Figure 1:**
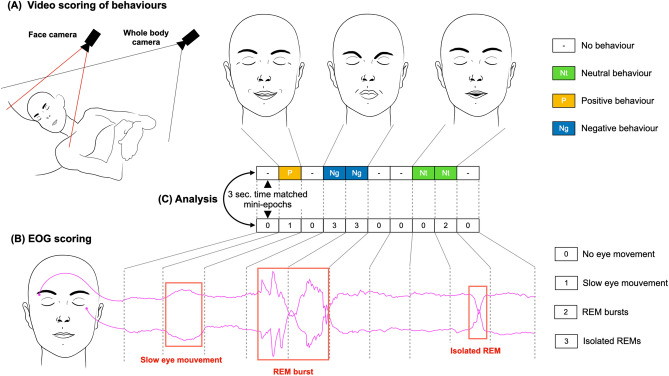
Schematic representation of the study methodology. (**A**) The video of the participants sleeping during REM sleep, filmed by two cameras, was cut into mini-epochs of 3 s. and visualized; each mini-epoch was classified as presenting an absence of behaviour, a behaviour without apparent emotion (qualified as neutral), a positive behaviour (smile, laughter) or a negative behaviour (painful, angry or sad faces, screaming, yelling at someone, aggressive behaviour, fighting …). (**B**) The electro-oculographic recording was divided into 3 s. mini-epoch time locked with the video and each mini-epoch was classified as without eye movements (EMs), with slow eye movements (SEMs), with isolated rapid eye movements (REMs) (1 or 2 consecutive REMs) or with REM bursts (3 or more consecutive REMs). (**C**) The association between the different types of EMs and the different behaviours was then analysed statistically.

The video-polysomnographic recordings during REM sleep of participants with RBD were divided into 3 s time intervals (mini-epochs) and assessed individually. Each mini-epoch was classified as either without observable behaviour, or with neutral, positive (i.e. smiling, laughing) or negative behaviour (i.e. shouting, fighting, yelling at someone, displaying painful face expressions). In parallel, the electro-oculographic (EOG) recordings of the participants were also visualized during the same time interval and classified as without EMs, with slow eyes movements (SEMs), with isolated REMs or with REM bursts. Associations between the observed behaviours and the type of EMs were then analysed. A schematic summary is presented in Fig. 1.

### Demographic and polysomnographic measures

The demographic and sleep parameters of participants are shown in Table 1. Of the 20 participants, 15 had idiopathic RBD, while 5 had RBD associated with Parkinson’s disease. All patients with Parkinson’s disease were treated with dopaminergic agents.

**Table 1:** Demographic and polysomnographic measures in participants with rapid eye movement sleep behaviour disorder (RBD).

	Participants with RBD
Number	20
Sex, female No. (%)	3 (15)
Idiopathic RBD, No. (%)	15 (75)
Parkinson’s disease, No. (%)	5 (25)
Age, years	65.1 ± 6.8
Total sleep time (TST), min	421.9 ± 80.2
Total sleep period (TSP), min	493.5 ± 91.8
Sleep efficiency (TST/TSP), %	85.7 ± 6.6
Sleep onset latency, min	22.1 ± 15.9
REM sleep latency, min	131.9 ± 78.8
**Sleep stages, min (% of TST)**	
NREM sleep stage N1	12.5 ± 6 (3.1 ± 1.8)
NREM sleep stage N2	206 ± 81 (48 ± 14.7)
NREM sleep stage N3	96.4 ± 42 (24 ± 11.3)
REM sleep	97.9 ± 45.6 (23.2 ± 9.5)
Arousals/hour of sleep	10.9 ± 7.8
Periodic leg movements/ hour of sleep	17.7 ± 24.3
Apnoea-hypopnea/hour of sleep	5.7 ± 7.6
REM sleep without atonia, % of REM sleep	65.7 ± 20.4

*RBD* rapid eye movement sleep behaviour disorder, *NREM* non rapid eye movement sleep.

### Behavioural measures

A total of 46 REM sleep episodes were analysed, totalling 20.7 h of REM sleep. Measures of behaviours are shown in Table 2. Behaviours occurred in 23.6% of the analysed REM sleep. Of these behaviours, 8.3% were emotional (5.1% positive, 3.2% negative) and 91.7% were neutral. Among the participants, 17 (85%) expressed at least one emotional behaviour. Expressions of negative emotions occurred in 14 (70%) and of positive emotions in 16 (80%). There was no difference in the proportion of positive and negative behaviours expressed by participants (p = 0.2). Among the behaviours, 4.6% were classified as major (N = 256/5854); versus 95.6% of behaviours classified as minor.

**Table 2:** Behavioural and electro-oculographic measures during REM sleep.

	Total mini-epochs (No.)	Total time (min)	Mini-epochs per subjects (No.)	Time per subject (min)
Video-polysomnography analysed	24,790	1,239.5	1240 ± 594	62 ± 30
Behaviours (all types)	5,854	292.7	293 ± 229	15 ± 11
Neutral emotional behaviours	5,370	268.5	269 ± 214	13 ± 11
Positive emotional behaviours	295	14.8	15 ± 20	1 ± 1
Negative emotional behaviours	189	9.5	9 ± 14	0.5 ± 0.7
Analysed EOG (without artifacts)	24,007	1200.3	1200 ± 574	60 ± 29
Without EMs	18,558	927.9	928 ± 467	46 ± 23
With slow EMs	956	47.8	48 ± 32	2 ± 2
With isolated REMs	2,337	116.9	117 ± 58	6 ± 3
With REMs bursts	2,156	107.8	108 ± 97	5 ± 5

*EOG* electro-oculography, *EMs* eye movements, *REMs* rapid eye movements.

### Measures of eye movements

Measures of EMs are shown in Table 2. The 3 s mini-epochs containing artifacts (N = 783/24,790 [3.2%]) were excluded. Artefacts were present in 1.9% of mini-epochs without behaviour, 6.1% of mini-epochs with neutral behaviour, 10.8% of mini-epochs with positive behaviour and 36.5% of mini-epochs with negative behaviour. Of the remaining mini-epochs, 77.3% contained no EMs, 4% contained SEMs, 9.7% contained isolated REMs and 9% contained REMs bursts.

### Association between eye movements and behaviours

**Figure 2:**
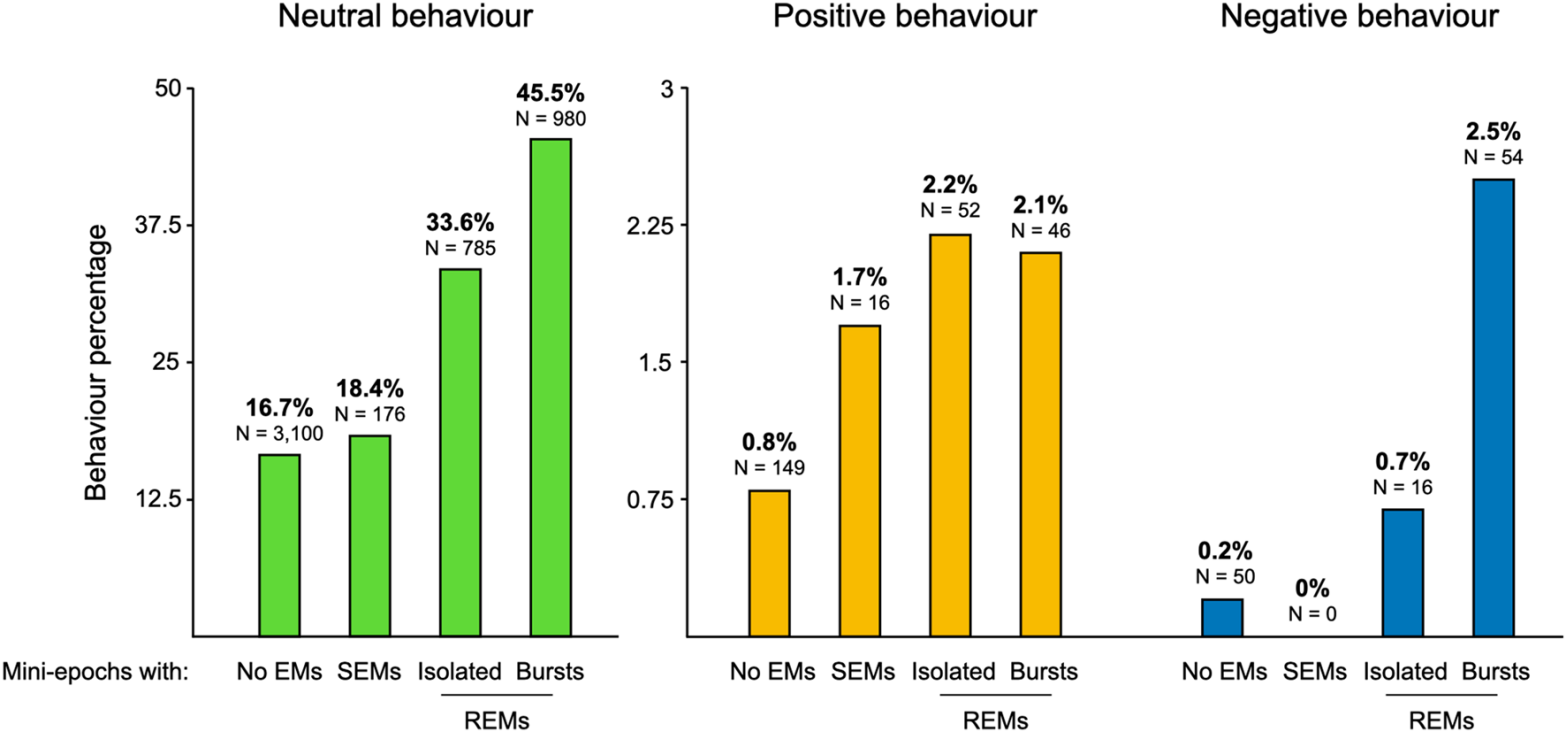
Percentage of mini-epochs with neutral, positive or negative emotional behaviours in conjunction with mini-epochs without eye movements (EMs), with slow eye movements (SEMs), isolated rapid eye movements (REMs) or REM bursts.

Among mini-epochs with behaviours, 60.8% were not associated with any concomitant eye movement, 35.6% were associated with REMs (15.7% with isolated REMs and 20% with REM bursts) and 3.6% were associated with SEMs. Associations between eye movements and behaviours are shown in Fig. 2. Statistical associations between types of EMs and behaviours are shown in Tables 3 and 4. A successive increase in the frequency of neutral behaviours was seen in mini-epochs with no EMs, SEMs, isolated REMs and REM bursts. Positive behaviours were more frequently observed in mini-epochs containing SEMs, isolated REMs and REM bursts relative to those without EMs. In contrast with neutral behaviours, there were no differences in positive behaviours when comparing mini-epochs containing isolated REMs or REM bursts with mini-epochs containing SEMs, nor when comparing mini-epochs containing REM bursts and mini-epochs containing isolated REMs. Negative behaviours were more frequently observed in mini-epochs containing isolated REMs and REM bursts relative to those without EMs and in mini-epochs containing REM bursts relative to those containing isolated REMs. No negative behaviours were observed in mini-epochs containing SEMs. The strongest association was found between REM bursts and negative behaviours. The same analysis contrasting different behaviour types relative to one another showed a stronger association between REM bursts and negative behaviours relative to positive and neutral behaviours. Slow eye movements had a stronger association with positive behaviours relative to neutral behaviours.

**Table 3:** Associations between eye movement types (contrasted with the absence of eye movements) and behavioural measures.

Dependent variables	Eye movements (EMs) effect (versus no EMs)					
	Slow EMs		Isolated REMs		REM bursts	
	OR [CI]	P	OR [CI]	P	OR [CI]	P
Neutral emotional behaviours	1.4 [1.2–1.6]	0.004	2.8 [2.5–3.1]	< 0.0001	4.6 [4.2–5.1]	< 0.0001
Positive emotional behaviours	2.8 [1.6–4.7]	0.001	2.8 [2–3.8]	< 0.0001	3 [2.1–4.2]	< 0.0001
Negative emotional behaviours	–	–	2.6 [1.4–4.5]	0.02	10.1 [6.7–15.1]	< 0.0001
Positive versus neutral emotional behaviours	2.4 [1.3–4.2]	0.03	1.4 [1–2]	> 0.05	1.1 [0.8–1.6]	> 0.05
Negative versus neutral emotional behaviours	–	–	1.3 [0.9–3.1]	> 0.05	3.5 [2.3–5.4]	< 0.0001
Negative versus positive emotional behaviours	–	–	1 [0.5–2]	> 0.05^a^	3.5 [2–6]	< 0.0001^a^

P values are presented after Bonferroni type corrections.

*EMs* eye movements, *SEMs* slow eye movements, *REMs* rapid eye movements, *OR* odds ratio, *CI* 95% confidence interval.

^a^A mixed logistic regression model could not be used because too few mini-epochs presented a combination of the contrasts of eye movements and behaviours used, a chi-square test was used.

**Table 4:** Contrast between eye movement types considering their association with behavioural measures.

Dependent variables	Isolated REMs vs. Slow EMs		REM bursts vs. Slow EMs		REM bursts vs. Isolated REMs	
	OR [CI]	P	OR [CI]	P	OR [CI]	P
Neutral behaviours	2 [1.6–2.4]	< 0.0001	3.3 [2.8–4.1]	< 0.0001	1.6 [1.4–1.8]	< 0.0001
Positive behaviours	1 [0.6–1.8]	> 0.05	1.1 [0.6–2]	> 0.05	1.1 [0.7–1.6]	> 0.05
Negative behaviours	–	–	–	–	4.2 [2.4 – 7.6]	< 0.0001
Positive versus neutral behaviours	0.6 [0.3–1.1]	> 0.05	0.5 [0.3–0.9]	> 0.05	0.8 [0.5–1.2]	> 0.05
Negative versus neutral behaviours	–	–	–	–	2.7 [1.5–5.1]	0.03^a^
Negative versus positive behaviours	–	–	–	–	3.8 [1.8–8.1]	0.003^a^

P values are presented after Bonferroni type corrections.

*SEMs* slow eye movements, *REMs* rapid eye movements, *OR* odds ratio, *CI* 95% confidence interval.

^a^A mixed logistic regression model could not be used because too few mini-epochs presented a combination of the contrasts of eye movements and behaviours used, a chi-square test was used.

The proportion of major behaviours was, in conjunction with the absence of REMs of 3.2% (N = 107/3299), 2.6% with SEMs (N = 5/192), 4.3% with isolated REMs (37/853) and finally 7.1% with the REM bursts (N = 77/1080). A logistic regression model analysing the association of the type of EMs and the proportion of major behaviours was significant (p < 0.0001) with in post hoc analysis a significant contrast between REM bursts and the absence of EMs (p < 0.0001; OR = 2.3 [1.7–3.1]; p > 0.05 for all other contrasts; Bonferroni post hoc correction). In addition, major behaviours were overrepresented in conjunction with negative behaviours (15%, N = 29/160) compared to neutral (4%, N = 216/5154) and positive (6%, N = 20/275) behaviours (p value of the logistic regression model < 0.0001; negative versus neutral post hoc contrast: p < 0.0001; negative versus positive: p = 0.009; positive versus neutral: p > 0.05; all with Bonferroni post hoc correction). Major movements were thus overrepresented in conjunction with negative behaviours on the one hand and REM bursts on the other hand. To exclude confusion between the association of REM bursts and major movements instead of REM bursts and negative emotion (versus neutral and positive behaviour) per se, the contrasts involving the REM bursts and negative emotions were relaunched in using major (versus minor) behaviours as an additional regressor in the analysis. The negative versus neutral contrast behaviours was still associated with the contrast between the REM bursts versus the absence of EMs (p < 0.0001; OR = 3.2 [2.1–4.7]) and REM burst versus isolated REMs (p = 0.03; OR = 2.6 [1.5–4.7]) as well as the contrast between negative versus positive behaviours with the contrasts between REM bursts versus no EM (p = 0.0002; OR = 3.2 [1.9–5.4]) and REM bursts versus isolated REMs (p = 0.01; OR = 3.6 [1.8–7, 3]) (all the p values are presented with the same level of Bonferroni correction as the initial contrasts).

## Discussion

In our study, REMs (isolated and in bursts), were temporally associated with both emotional and neutral behaviours in participants with RBD. The strongest association was between negative emotional behaviours and REM bursts. Slow eye movements were also associated with positive and neutral behaviours but were absent during negative behaviours.

Associations between REMs and motor manifestations of RBD have previously been reported in 3 studies. Frauscher et al.^22^ found more complex, major and violent movements during phasic (when REMs occur) than tonic 30 s epochs of REM sleep in patients with RBD. Manni et al.^23^ found that REMs were associated with more primitive, purposeful and semi purposeful motor behaviours, as well as vocalizations. Leclair-Visonneau et al.^24^ found that all types of behaviours were more frequent during 30 s epochs containing REMs than those without REMs. As in previous studies, we observed an association between eye movements and behaviours, without however the association being systematic since most observed behaviours (60.8%) do not coincide with eye movements. In the present study, we contribute to this field by extending our assessment for the first time to SEMs, by distinguishing between isolated REMs and REM bursts and by examining the relationship between eye movements and the emotional valence of behaviours.

Although both isolated REMs and REM bursts are associated with behaviours in RBD, they display two distinct association profiles. Neutral behaviours are more strongly associated with REM bursts than isolated REMs. Isolated REMs and REM bursts are equally associated with positive behaviours, whereas the association with negative behaviours is stronger for REM bursts. These results suggest that isolated REMs are non-preferentially associated with both positive and negative emotions whereas REM bursts are more strongly associated with negative than positive emotions.

This result suggest that isolated REMs and REMs bursts have different meanings. Differences between isolated REMs and REM bursts have been highlighted by several approaches. Twin studies have shown that REM bursts are more heritable than isolated REMs^25^. REMs bursts (unlike isolated REMs) can be experimentally induced by injecting acetylcholine in sleeping cats’ brainstem^26^. Our results expand this literature by demonstrating a difference in the behavioural associations of these different REM types. Interestingly, in new-borns (in whom REM sleep is not accompanied by atonia) smiles are over-represented during periods containing abundant REM bursts but not in those containing isolated REMs. In cats with experimentally induced RBD, bursts of REMs are associated with attack behaviours, which could be classified as negative behaviours^27^.

An association between SEMs and specific behaviours in participants with RBD has never, to our knowledge, been demonstrated. Slow eye movements were here most associated with positive behaviours, and never occurred during negative behaviours. Therefore, SEMs appear to exhibit greater specificity for an association with positive emotions and could become a potential marker of positive emotion during REM sleep. If true, it would be interesting to study whether SEMs density (percentage of REM sleep with SEMs) is decreased in subjects suffering from mental disorders (notably, depression).

Although our findings are preliminary, it is interesting to consider them in relation to previous hypotheses with regards to the association between eye movements and the emotional content of dreams. According to the “scanning” hypothesis, REMs are suggested to correspond to saccadic visual exploration of dream imagery. One may imagine that negative emotional scenes are more chaotic in nature, and would stimulate a more intense visual exploration. The greater association that we observed between negative emotional scenes and REM bursts may support this intuition. An alternate (though not mutually exclusive) theory has been proposed that REMs are involved in the emotional regulation thought to occur in REM sleep. Stickgold has suggested that eye movements in REM sleep produce an attentional shift concomitant with reactivation of negative memories, in a manner similar to eye movement desensitization and reprocessing (EMDR) therapy, but performed during sleep rather than wakefulness^4^. Indeed, EMDR is a psychotherapy treatment designed to alleviate the distress associated with traumatic memories^28^. During therapist-directed lateral eye movements, patients recall traumatic events, which is supposed to facilitate the accessing of the traumatic memory network, so that information processing is enhanced, with new associations forged between the traumatic memory and more adaptive memories or information^29^. The stronger association found between negative behaviours and REM bursts could also support the hypothesis that our brain practices its own EMDR during REM sleep, as a mechanism to desensitize negative emotions. Further studies assessing the impact of REM bursts density in populations of healthy subjects or with psychiatric disorders could provide a better understanding of their possible function in emotional regulation.

We acknowledge some limitations in our study. Our sample was relatively small, and patients did not contribute equally to the number of analysed REM sleep episodes. This reduced sample was balanced by a large (N = 24,790) number of visually studied mini epochs. The sample was mixed, as some patients had idiopathic RBD and others had RBD associated with Parkinson’s disease. Bugalho et al.^30^ found no difference in the frequency and pattern of motor events (including negative and positive emotional behaviours) during REM sleep on videopolysomnography in 14 patients with Parkinson's disease and RBD (all treated with dopaminergic drugs) vs. 18 patients with idiopathic RBD (not treated with dopaminergic drugs), suggesting that Parkinson's disease and treatment with dopaminergic agents have no major impact on the behaviours of patients with RBD. Eventually, the research was focused on the EOG correlates of emotions during REM sleep, whatever the dreaming content and its influences (previous day experiences, mood, drugs…). Moreover, we minimized the variability due to inter-subject differences with a logistic mixed-model analysis. Plus, these disorders are close, as idiopathic RBD later evolves in Parkinson’s disease. Most patients slept under sheets, thus, we selected only video sequences with visible faces and upper arms. We did not awaken sleepers to collect their self-reported dream emotion. Consequently, the evaluation of emotions was exclusively external. The goal of our study, however, was to bypass the inherent problems with dream reports entirely. Double scoring was done for a sample of the videos with a good concordance rate between scorers. The main limitation of our study is that RBD is a pathological model, which may limit the generalisability of our findings to healthy subjects. However, REMs during REM sleep do not differ in type or frequency between healthy subjects and those with RBD^24^. Moreover, dream content, including emotional content, does not differ between patients with RBD and healthy subjects^31^. We therefore hypothesize that associations between eye movement and dream content could be extended to healthy subjects, without completely excluding that the association itself between dreams and EMs could be altered in the RBD model.

Our study supports an association between REMs, but also SEMs, and emotions during REM sleep. Indeed, SEMs are associated with positive but not with negative emotions, REMs are associated with both positive and negative emotions, and REM bursts have the strongest association with negative behaviours, suggesting a role of REM bursts in the processing of negative emotions during sleep. The RBD model allows partial, but direct access to the emotion of the ongoing dream and could make it possible in the future to highlight other associations between emotions and polysomnogaphic parameters such as EEG (with the limit of artefacts caused by participants behaviour).

## Methods

### Participants

Twenty consecutive participants with RBD were included in this study. They were taking part in a larger study on prodromal (idiopathic RBD) and early phases of Parkinson’s disease (ICEBERG trial, clinicaltrial.gov: NCT02305147). All the participants were informed of the objectives of the study and signed a written informed consent including the sleep and video study. The study was approved by the local ethics committee (CPP Paris VI/RCB: 2009-A00922-55). All methods were conducted in strict accordance with relevant guidelines and regulations, including the ethical standards set forth in the 1964 Declaration of Helsinki and its subsequent amendments. RBD was diagnosed using the International Classification of Sleep Disorders-3 diagnostic criteria^32^. Patients had idiopathic RBD (no concomitant neurological disorder, as assessed by neurologists), or RBD associated with Parkinson’s disease. Polysomnography was performed at the Sleep Disorders Unit of the Pitié-Salpêtrière hospital between May 2016 and April 2018. Subjects treated with antidepressants or benzodiazepines or any other psychotropic drug except dopaminergic treatments were not included in the study. All patients were interviewed by a psychiatrist, and only one suffered of an untreated depression at time of polysomnography.

### Sleep evaluation

All patients underwent video-polysomnography for one attended night. The monitoring included Fp1-A2, C3-A2, C3-O1 electroencephalography (EEG), mentalis and right and left tibialis anterior muscle surface electromyography, nasal pressure through nasal prongs, naso-oral thermistor, recording of tracheal sounds through a microphone placed at the surface of the trachea, thoracic and abdominal plethysmography to assess respiratory efforts, electrocardiography, pulse oximetry, EEG-synchronized infrared video-monitoring and ambiance microphone recordings. The video monitoring included two different cameras, one allowing a general view on the sleeper’s full body and a second focused on the face.

### Monitoring and classification of behaviours

We selected those REM sleep sequences where the participants face and upper arms were clearly visible on video monitoring. The videos were divided into and analysed in 3 s mini-epochs. We selected mini-epochs with observable behaviours containing continuous movements lasting as least 3 s without interruption, even if not obviously purposeful or scenic. The behaviours were qualified by the raters as positive (presence of bilateral smiling and laughs as described in Clé et al.)^20^ and negative (pouting, shouts, and behaviours suggestive of pain or painful experiences, sadness, fear or anger as described in Maranci et al.)^21^, continuous movements of participants that did not show obvious signs of positive or negative emotions were defined, by default, as neutral. All video clips were scored by a single rater (JB.M), and 11% of them were double watched by a second scorer (EF.M). The inter-rater agreement was 85%, the Cohen’s Kappa score was 0.68. Since Kappa is highly dependent on prevalence^33^ (because Kappa values may under estimate the validity of the scoring when rare events are present), we calculated a Prevalence-Adjusted and Bias-Adjusted Kappa (PABAK) of 0.8. Plus, all mini epochs classified as emotional by the first scorer were systematically double watched by a third scorer (MC.V), 98% were scored as the same emotion, for the remaining 2% (N = 12) the emotion was deemed ambiguous and they were discarded from the analysis.

In addition to this classification based on emotion, all of the mini-epochs with behaviours were classified by considering the amplitude of the movements as major behaviour (carrying out large movements such as raising the arms or lifting the leg) or by default as minor behaviour (as described in previous studies^22,34^, but excluding major "jerks" in our own study since only the continuous behaviours suggestive of a dreamlike activity interested us). Only one scorer (JB. M) achieved this classification.

### Assessment of eye movements

REMs were defined as synchronized changes of EOG potentials of opposing polarity in the two EOG channels, lasting less than 0.5 s and exceeding the threshold of 25 microvolts^35^. We defined REMs bursts as a sequence of three or more consecutive REMs separated by less than 2 s^25^. Non-consecutive REMs were classified as isolated REMs. Slow eye movements (SEMs) were defined as conjugate, rolling eye movements with an initial deflection ≥ 0.5 s in duration^36^. To assess the temporal association between behaviours and eye movements during REM sleep, we defined 3 s mini-epochs in the EOG channel. Each mini-epoch was visually classified as: without eyes movement (no REMs or SEMs); with SEMs; with isolated REMs; or with REM bursts. All EOG scoring was done by a single rater (JB.M), and 11% of them were scored by a second rater (C.C). The inter-rater agreement was 92%, the Cohen’s Kappa was 0.78, the PABAK was 0.9. Behavioural and eye movements classifications were time matched, and the two classifications compared within the same mini-epoch timeframe.

### Statistical analysis

To test the association between the different types of EMs and the different types of behaviours, we used the 3 s mini-epochs described above as the unit of analysis. Because the distribution of behaviours differed between subjects, we used a mixed logistic regression model with the lme4 package in R so as to minimize the impact of inter-individual variability^37^. The type of behaviour was used as a dependent variable, the type of EMs as an independent variable with a fixed effect, and the identity of the subjects as a random independent variable. The effect of any EMs on behaviour was first evaluated by contrasting them with the absence of EMs, Bonferroni corrections were applied. Subsequently, the different types of EMs were contrasted with each other, with additional Bonferroni corrections. Regarding dependent variables, contrasts between behaviours were also applied. Some combinations of dependent and independent variable contrasts were too infrequent to apply a mixed model to. In these cases, a chi-square test was used as specified in the different sections of the results. The association between major behaviours and the type of EMs on the one hand and the association with emotional valence on the other hand was analysed with a logistic regression model (not mixed) with the amplitude of the behaviour (major versus minor) as a dependent variable and either the type of EMs or the valence as regressor. Once the association between the amplitude of the behaviours and their valence as well as the type of EMs was confirmed, the different relevant contrasts previously launched were re-launched with the amplitude of behaviours (major/minor) as a supplemental dependent variable (non-mixed model). The prevalence of emotions within subjects was compared with a Wilcoxon test. The statistical software used was R^38^.

## References

[1] Palagini L, Baglioni C, Ciapparelli A, Gemignani A, Riemann D (2013). REM sleep dysregulation in depression: State of the art. Sleep Med. Rev..

[2] Pillai V, Kalmbach DA, Ciesla JA (2011). A meta-analysis of electroencephalographic sleep in depression: Evidence for genetic biomarkers. Biol. Psychiat..

[3] Arnulf I (2011). The “scanning hypothesis” of rapid eye movements during REM sleep: A review of the evidence. Arch. Ital. Biol..

[4] Stickgold R (2002). EMDR: A putative neurobiological mechanism of action. J. Clin. Psychol..

[5] Harrington MO, Pennington K, Durrant SJ (2017). The ‘affect tagging and consolidation’ (ATaC) model of depression vulnerability. Neurobiol. Learn. Mem..

[6] Walker MP, van der Helm E (2009). Overnight therapy? The role of sleep in emotional brain processing. Psychol. Bull..

[7] Miyauchi S, Misaki M, Kan S, Fukunaga T, Koike T (2009). Human brain activity time-locked to rapid eye movements during REM sleep. Exp. Brain Res..

[8] Hori T, Ogawa K, Abe T, Nittono H (2008). Brain potentials related to rapid eye movements and dreaming during REM sleep: A short review of psychophysiological correlates. Sleep Biol. Rhythms.

[9] Ogawa K, Abe T, Nittono H, Yamazaki K, Hori T (2009). Temporal coupling of rapid eye movements and cerebral activities during REM sleep. Clin. Neurophysiol..

[10] Ioannides AA (2004). MEG tomography of human cortex and brainstem activity in waking and REM sleep saccades. Cereb. Cortex.

[11] Corsi-Cabrera M (2016). Human amygdala activation during rapid eye movements of rapid eye movement sleep: An intracranial study. J. Sleep Res..

[12] Andrillon T, Nir Y, Cirelli C, Tononi G, Fried I (2015). Single-neuron activity and eye movements during human REM sleep and awake vision. Nat. Commun..

[13] Janak PH, Tye KM (2015). From circuits to behaviour in the amygdala. Nature.

[14] Armony JL (2013). Current emotion research in behavioral neuroscience: The role(s) of the amygdala. Emot. Rev..

[15] Hong C, Harris J (2009). fMRI evidence for multisensory recruitment associated with rapid eye movements during sleep. Hum. Brain Mapp..

[16] De Carli F (2016). Activation of the motor cortex during phasic rapid eye movement sleep. Ann. Neurol..

[17] Blake Y, Terburg D, Balchin R, van Honk J, Solms M (2019). The role of the basolateral amygdala in dreaming. Cortex.

[18] Schenck CH, Bundlie SR, Ettinger MG, Mahowald MW (1986). Chronic behavioral-disorders of human rem-sleep—a new category of parasomnia. Sleep.

[19] Valli K (2012). Can observers link dream content to behaviours in rapid eye movement sleep behaviour disorder? A cross-sectional experimental pilot study. J. Sleep Res..

[20] Clé M (2019). Smiling asleep: A study of happy emotional expressions during adult sleep. J. Sleep Res..

[21] Maranci J-B, Aussel A, Vidailhet M, Arnulf I (2021). Grumpy face during adult sleep: A clue to negative emotion during sleep ?. J. Sleep Res..

[22] Frauscher B (2009). The relation between abnormal behaviors and REM sleep microstructure in patients with REM sleep behavior disorder. Sleep Med..

[23] Manni R, Terzaghi M, Glorioso M (2009). Motor-behavioral episodes in REM sleep behavior disorder and phasic events during REM sleep. Sleep.

[24] Leclair-Visonneau L, Oudiette D, Gaymard B, Leu-Semenescu S, Arnulf I (2010). Do the eyes scan dream images during rapid eye movement sleep? Evidence from the rapid eye movement sleep behaviour disorder model. Brain.

[25] Adamczyk M (2015). Genetics of rapid eye movement sleep in humans. Transl. Psychiatry.

[26] Jeannerod M, Mouret J, Jouvet M (1965). Étude de la motricité oculaire au cours de la phase paradoxale du sommeil chez le chat. Electroencephalogr. Clin. Neurophysiol..

[27] Soh K, Morita Y, Sei H (1992). Relationship between eye-movements and oneiric behavior in cats. Physiol. Behav..

[28] Shapiro F (1989). Efficacy of the eye movement desensitization procedure in the treatment of traumatic memories. J. Trauma. Stress.

[29] Shapiro F, Maxfield L (2002). Eye movement desensitization and reprocessing (EMDR): Information processing in the treatment of trauma. J. Clin. Psychol..

[30] Bugalho P (2017). Characterization of motor events in REM sleep behavior disorder. J. Neural Trans..

[31] D’Agostino A (2012). Challenging the myth of REM sleep behavior disorder: No evidence of heightened aggressiveness in dreams. Sleep Med..

[32] Sateia MJ (2014). International classification of sleep disorders-third edition. Chest.

[33] Chen G, Faris P, Hemmelgarn B, Walker RL, Quan H (2009). Measuring agreement of administrative data with chart data using prevalence unadjusted and adjusted kappa. BMC Med. Res. Methodol..

[34] Oudiette D (2012). A motor signature of REM sleep behavior disorder. Movem. Disord..

[35] Takahashi K, Atsumi Y (1997). Precise measurement of individual rapid eye movements in REM sleep of humans. Sleep.

[36] Malhotra RK, Avidan AY (2014). Sleep stages and scoring technique. Atlas of Sleep Medicine.

[37] Bates D, Mächler M, Bolker BM, Walker SC (2015). Fitting linear mixed-effects models using lme4. J. Stat. Softw..

[38] R Development Core Team. R: A Language and Environment for Statistical Computing. http://www.r-project.org/10.1007/978-3-540-74686-7.

